# Prefrontal cortex functional connectivity changes during verbal fluency test in adults with short-term insomnia disorder: a functional near-infrared spectroscopy study

**DOI:** 10.3389/fnins.2023.1277690

**Published:** 2023-11-09

**Authors:** Peirong Wu, Chaowen Wang, Mindong Wei, Yijiang Li, Yuan Xue, Xinrong Li, Jianfan Jiang, Yinuo Bi, Jian Dai, Wenyu Jiang

**Affiliations:** ^1^Department of Neurological Rehabilitation, Jiangbin Hospital of Guangxi Zhuang Autonomous Region, Nanning, China; ^2^Cognitive Rehabilitation Center, Jiangbin Hospital of Guangxi Zhuang Autonomous Region, Nanning, China; ^3^Faculty of Science and Engineering, University of Nottingham Ningbo, Ningbo, China; ^4^Department of Clinical Psychology, Jiangbin Hospital of Guangxi Zhuang Autonomous Region, Nanning, China

**Keywords:** functional near-infrared spectroscopy, short-term insomnia disorder, verbal fluency test, functional connectivity, prefrontal cortex

## Abstract

**Background:**

Individuals suffering from short-term insomnia disorder (SID) experience difficulties in falling or staying asleep, often leading to daytime fatigue and impaired concentration. However, the underlying mechanisms of SID remain unclear. This study aims to investigate the alterations in brain activation patterns and functional connectivity in patients with SID.

**Methods:**

The study enrolled a total of 31 adults diagnosed with SID and 31 healthy controls (HC). Functional near-infrared spectroscopy (fNIRS) was utilized to assess the concentrations of oxyhemoglobin (Oxy-Hb) and functional connectivity in the prefrontal cortex of each participant while performing the verbal fluency test (VFT) task.

**Results:**

In the VFT task, no significant difference was found between the SID group and the HC group in terms of integral values, centroid values, and mean Oxy-Hb variations. These findings suggest that both groups exhibit similar hemodynamic responses. However, the functional connectivity analysis revealed significant differences in inter-channel connectivity strength between the two groups. The SID group showed significantly lower average inter-channel connectivity strength compared to the HC group. Moreover, six channel pairs (right frontopolar cortex – left frontopolar cortex, left orbitofrontal cortex – left temporopolar cortex, left temporopolar cortex – left frontopolar cortex, left frontopolar cortex-Ch38, left frontopolar cortex – right pre-motor and supplementary motor cortex, and left frontopolar cortex – right dorsolateral prefrontal cortex) exhibited significantly higher connectivity strength in the HC group compared to the SID group (FDR corrected, *p* < 0.05). Specifically, channel 27 exhibited the highest frequency of significant connectivity across different channel pairs, occurring five times in total. The channel pair Ch27-Ch39, representing left frontopolar cortex and right dorsolateral prefrontal cortex, exhibited a negative correlation with PSQI scores (r = −0.422, *p* = 0.018).

**Conclusion:**

Our findings suggest that patients with SID may exhibit altered brain connectivity during the VFT task, as measured by fNIRS. These results provide valuable insights into the functional brain differences associated with SID. Further research is needed to validate and expand upon these findings.

## 1. Introduction

According to a White paper on healthy sleep published on the internet in 2023, over 60% of the Chinese population suffers from sleep-related issues. Insufficient sleep has been associated with memory decline, weakened immunity, negative emotions, and increased risk of endocrine disorders and cardiovascular diseases (Bin Heyat et al., 2021). Insomnia, a prevalent sleep disorder, is characterized by difficulties falling asleep, sleep maintenance problems, early waking, and impaired daytime functioning (Patel et al., 2018). Short-term insomnia disorder (SID) is diagnosed when symptoms persist for less than 3 months (Vargas et al., 2020). Approximately 27% of the population experiences SID annually, with about 72% recovering without any treatment. However, a subset of people with persistent symptoms progress to chronic insomnia disorder (CID; LeBlanc et al., 2009). Despite sharing similar clinical symptoms, SID and CID exhibit distinct pathophysiological bases and clinical characteristics, such as etiology, incidence, symptom severity. While SID arises from acute hyperarousal, CID is more likely due to prolonged exposure to hyperarousal and poor sleep patterns, leading to a “conditioned” arousal (Vargas et al., 2020). Thus, SID and CID should not be considered different stages of a single disease, and CID should not be solely explained by a single pattern of hyperarousal. Therefore, studying SID contributes to a deeper understanding of its unique pathophysiological basis and clinical characteristics, in order to provide more individualized and effective interventions for this condition, and to avoid simply regarding SID as an early stage of CID, thus offering better treatment and management strategies for patients.

Investigators are increasingly focusing on the neuropathological mechanism of insomnia and tailoring clinical treatments accordingly. Neuroimaging studies have demonstrated that the prefrontal cortex (PFC) as a critical brain region involved sleep regulation and maintenance (Perrier et al., 2015; Anastasiades et al., 2022). The PFC becomes progressively deactivated during the transition from wakefulness to non-rapid eyes movement (NREM) sleep, and this deactivation intensifies with deeper NREM sleep, subsequently reactivating during sleep (Muzur et al., 2002). Moreover, sleep deprivation has been found to impair brain network function in the PFC, highlighting its vulnerability to insomnia (Mukli et al., 2021). Previous neuroimaging studies have shown that individuals with primary insomnia exhibit the following: (1) increased overall brain metabolism during wakefulness and sleep (Nofzinger et al., 2004), (2) decreased relative metabolism in the PFC compared to other brain region during wakefulness (Nofzinger et al., 2004), and (3) reduced PFC response during verbal fluency test (VFT) task compared to healthy individuals (Gong et al., 2022).

The VFT task is a widely utilized assessment tool for evaluating language and executive control abilities in individuals with neurological and psychiatric disorders (Xu et al., 2023). These abilities are closely linked to fundamental cognitive functions such as working memory, motivation, and attention. Published NIRS studies have demonstrated that patients with CID exhibit abnormal prefrontal activation during the VFT task (Sun et al., 2017; Gong et al., 2022). Based on this evidence, it is logical to hypothesize that the cognitive decline observed in individuals with insomnia is associated with dysfunction in the prefrontal cortex (PFC). Consequently, the integration of NIRS and the VFT task allows for the assessment of PFC function and identification of cognitive impairment in individuals with insomnia.

However, the majority of research conducted on insomnia thus far has primarily focused on patients with CID, whereas less attention has been directed towards SID and its underlying mechanisms remain unclear. In recent years, functional near-infrared spectroscopy (fNIRS), a non-invasive optical imaging technique used to monitor cerebral cortex oxygenation and dynamics, has been extensively employed to observe brain activity patterns in various diseases, leading to a better understanding of the mechanisms underlying neuropsychiatric impairments. Compared to other neuroimaging methods, fNIRS offers the advantages of portability, cost-effectiveness, and relative insensitivity to movement, making it suitable for assessing brain activity in different environments. By utilizing fNIRS to evaluate of PFC activity in individuals suffering from SID, it may be possible to explore the underlying mechanisms of SID and provide valuable insights for clinical interventions.

The present study aims to utilize fNIRS to investigate the changes in PFC activation and functional connectivity in patients with SID during a verbal fluency test (VFT) task. Additionally, the study seeks to explore the relationship between these alterations and impaired sleep quality.

## 2. Materials and methods

### 2.1. Participants

For this research, a total of 31 patients with SID and 31 gender-, age-and education-matched healthy controls (HC) were recruited from the community. The sleep quality of all participants in the past month was assessed using the Pittsburgh Sleep Quality Index (PSQI). Cognitive status was evaluated using the Montreal Cognitive Assessment (MoCA), while anxiety and depression levels were measured using the Hamilton Anxiety Rating Scale (HAMA) and Hamilton Depression Rating Scale (HAMD_24_), respectively.

The inclusion criteria for the SID group were as follows: (1) meeting the diagnostic criteria of short-term insomnia disorder outlined in third edition of the International Classification of Sleep Disorders (ICSD-3; Medicine AAoS, 2014), with insomnia occurring at least 3 days per week for a duration of 1 week to 3 months; (2) PSQI score > 7 (Buysse et al., 1989); (3) MoCA score > 26; (4) right-handedness. The exclusion criteria included: (1) taking neuropsychiatric drugs within past 3 months; (2) presence of cognitive impairment, emotional disorders, neuropsychiatric disorders, or any serious physical illnesses. Control volunteers with similar social backgrounds and PSQI scores ≤7 were enrolled, except for left-handers and individuals with neurological or psychiatric illnesses.

The study protocol was approved by the Ethics Committee of Jiangbin Hospital of Guangxi Zhuang Autonomous Region. All individuals involved in the study were fully informed about the study and provided written informed consent.

### 2.2. Verbal fluency test

**Figure 1:**
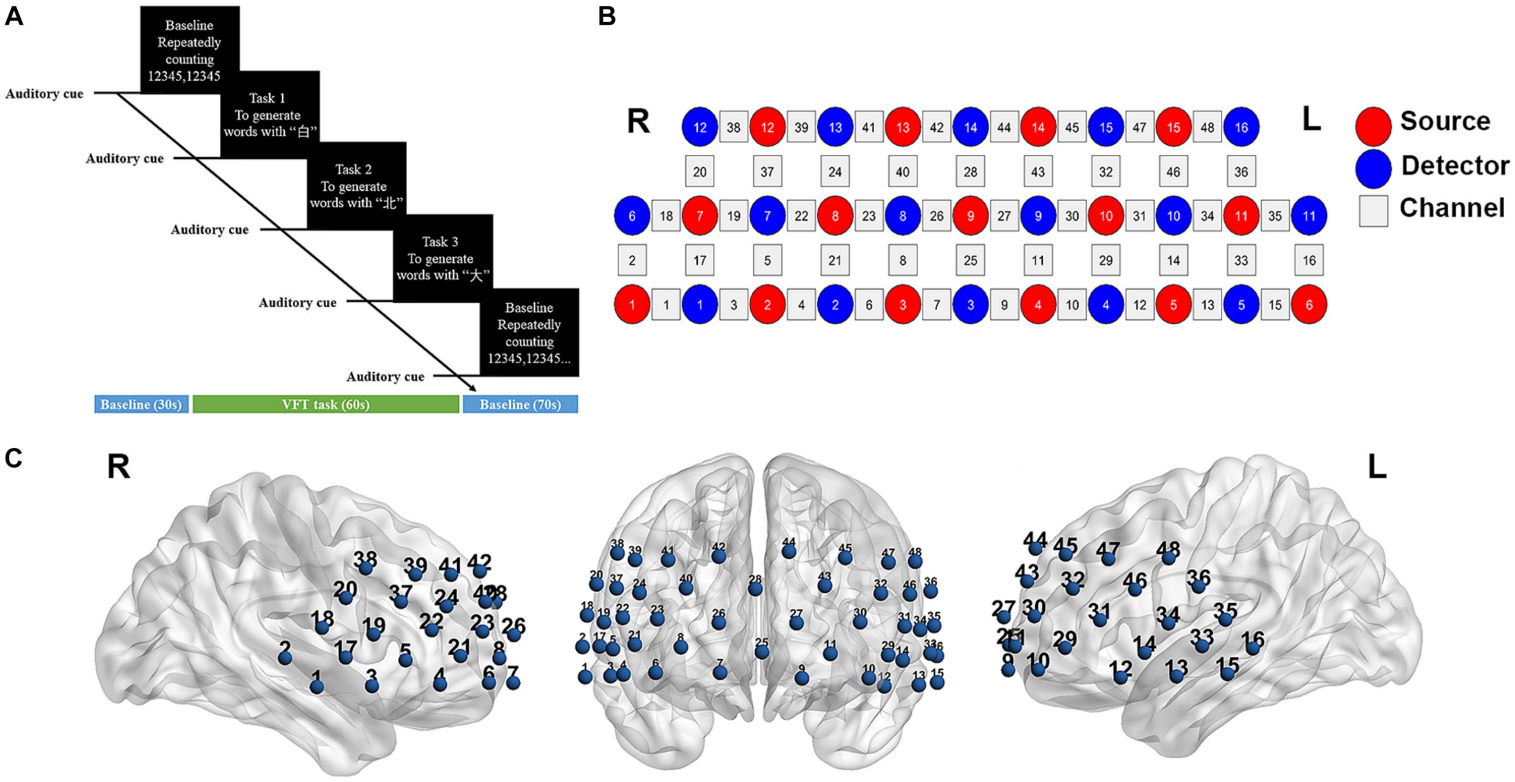
fNIRS data acquisition. **(A)** The task procedure of verbal fluency test (VFT). **(B)** Measurement points of the 48-channel NIRS system. Red and blue circles represent light source and detector, respectively. Gray squares represent the location of each probes pair (channel). **(C)** Three-dimensional detection regions of 48 channels. L, left; R, right.

The verbal fluency test (VFT) task was conducted to elicit the cerebral cortex activation. The entire test was conducted in a quiet and comfortable environment. Prior to initiating the formal test, all participants were provided with detailed instructions regarding the task procedure and were given an opportunity to practice. The VFT task consisted of three consecutive trials: a 30-s pre-task baseline, a 60-s task period, and a 70-s post-task baseline (as illustrated in Figure 1A). During the pre-task baseline, participants were instructed to repeatedly count from 1 to 5. In the task period, participants were required to generate as many words as possible using the Chinese characters “白” (representing white), “北” (representing north), and “大” (representing big). Lastly, during the post-task baseline, participants again repeated the act of counting from 1 to 5 repeatedly. To prevent periods of silence, the given three characters were changed every 20 s during the 60-s task period. The total number of correct words generated was recorded as a measure of task performance.

### 2.3. NIRS measurement

The changes in hemoglobin (Hb) concentration were measured using a multi-channel NIRS instrument (NirScan-6000C, Danyang Huichuang Medical Equipment Co., Ltd., China) that utilized laser diodes emitting two wavelengths (730 and 850 nm) during the VFT task. The sampling frequency was set at 11 Hz. A total of 31 probes (comprising 15 sources and 16 detectors) were positioned at a fixed distance of 3 cm. The placement of these probes followed the 10/20 international electrode placement system, with the FPz channel serving as the center of detector D3, and the lowest probes positioned along the Fp1-Fp2 line. Each pair of sources and detectors formed a channel, allowing for the observation of hemoglobin concentration information at a depth of 2–3 cm beneath the scalp. A total of 48 channels were created, symmetrically distributed across the bilateral PFC and temporal cortex of each participant (Figures 1B,C). The channels and corresponding brain areas are detailed in Supplementary Table 1.

### 2.4. Data processing and analysis

The processing of near-infrared spectroscopy data was conducted using NirSpark software (Huichuang, China), which has been used in previous research (Li et al., 2020). Preprocessing steps involved an initial inspection of the raw data by an expert to identify and discard poor-quality signals. Participants with more than five bad channels were excluded from further analysis. Motion artifacts were addressed using a spline interpolation algorithm, a commonly employed method for correcting localized artifacts. Motion artifacts typically manifest as sudden jumps or cliff-like changes caused by the relative displacement of the scalp and the probe (Sutoko et al., 2020). Physiological noise, such as respiration, cardiac activity, and low-frequency signal drift, were mitigated through band-pass filtering set between 0.01 and 0.1 Hz. The modified Beer–Lambert law was employed to calculate relative changes in both oxygenated hemoglobin (Oxy-Hb) and deoxygenated hemoglobin (Deoxy-Hb) concentrations (Xia et al., 2022). Among the two, Oxy-Hb has a higher signal-to-noise ratio and was analyzed as the primary index for observing hemodynamic changes (Hoshi et al., 2001).

The start time of the VFT task period was set as “0 s,” and the hemodynamic response function (HRF) was defined with an initial time of −10 s and an end time of 115 s (Liu et al., 2022). Specifically, “−10–0 s” represented the pre-baseline state, while “0–60s” corresponded to the duration of the block paradigm, and “60–115 s” represented the post-baseline period. For each channel, the integral value and centroid value were calculated to capture the time-course changes and quantitatively describe the waveform characteristics of the NIRS signal. The integral value indicated the magnitude of the signal changes during the 60s task period, while the centroid value represented the time point corresponding to half of the area under the positive change curve of the NIRS spectrum for the entire task period (from −10 to 115 s). Additionally, the average change in Oxy-Hb was analyzed for each channel during the 60s task period. By calculated hemoglobin concentration across all channels for each participant, we obtained the average hemoglobin concentration at the group level. Moreover, functional connectivity (FC) values were derived through Pearson correlation analysis of the time series data for each channel pair. And then Fisher’s r-to-z transformation was conducted for improving the normality (Li et al., 2023). This produced a correlation matrix of dimensions 48 × 48 for each participant.

### 2.5. Statistics analysis

Clinical data, neuropsychological test scores and VFT performance were analyzed using SPSS 17.0 (SPSS Inc., Chicago, IL, USA). Initially, the Kolmogorov–Smirnov test was employed to ascertain the normality of the quantitative data. Subsequently, inter-group comparisons were carried out based on the distribution of data: data that followed a normal distribution underwent statistical analysis using independent *t*-tests, while data with a non-normal distribution were assessed using the Mann–Whitney U test. The gender variable, treated as binary, was evaluated using the chi-squared test. Statistical significance was defined as *p* < 0.05.

The Near-infrared spectroscopy data was analyzed using NirSpark software. GraphPad Prism 8.0, BrainNetViewer and Photoshop CS6 were used for generating figures. A two-sample t-test was applied to compare the hemodynamic changes and FC values between the two groups, aiming to identify patterns brain activation and connection. False discovery rate (FDR) correction was applied to the fNIRS data. Statistical significance was defined as *p* < 0.05. Additionally, the mean Oxy-Hb changes or mean FC values were extracted from channels that exhibited statistical differences, and correlation analysis with PSQI scores was performed using Pearson’s method.

## 3. Results

### 3.1. Demographic and clinical characteristics

No significant difference was found in age, gender, years of education, VFT performance and MoCA total scores between the SID group and HC group (*p* > 0.05). However, the SID group exhibited significantly higher PSQI scores compared to the HC group (*p* < 0.05). Although the HAMA and HAMD scores of the SID group were also significantly higher than those of the HC group (*p* < 0.05), it is noteworthy that neither group met the criteria for clinical diagnosis of anxiety or depression. Please refer to Table 1 for a comprehensive summary of these findings.

**Table 1:** Comparison of clinical data and neuropsychological scores between the two groups.

Characteristics	SID group	HC group	*p* value
Gender (M/F)	12/19	15/16	0.609^a^
Age (years)	43.74 ± 15.61	43.19 ± 14.91	0.888
Education (years)	12.23 ± 3.47	11.63 ± 3.54	0.510
Duration (months)	1.88 ± 0.69	/	/
VFT performance	8.42 ± 2.23	9.26 ± 2.14	0.137
PSQI scores	10.03 ± 1.97	4.42 ± 2.14	<0.001
MoCA total scores	27.71 ± 1.37	27.84 ± 1.34	0.710
HAMA scores	1.13 ± 1.28	0.55 ± 0.85	0.040
HAMD_24_ scores	1.74 ± 1.63	0.48 ± 0.85	<0.001

SID, short-term insomnia disorder; HC, heathy control; VFT, verbal fluency test; PSQI, Pitsburgh sleep quality index; MoCA, Montreal Cognitive Assessment; HAMA, Hamilton Anxiety Rating Scale; HAMD_24_, Hamilton Depression Rating Scale. *p* < 0.05 indicates statistical significance. ^a^χ^2^ test for sex (*n*). Independent t-test for the other data (means ± SD).

### 3.2. Hemodynamic response during the VFT task

During the VFT task, no significant difference was observed in the integral values, centroid values or mean Oxy-Hb changes between the two groups (*p* > 0.05, FDR corrected).

**Figure 2:**
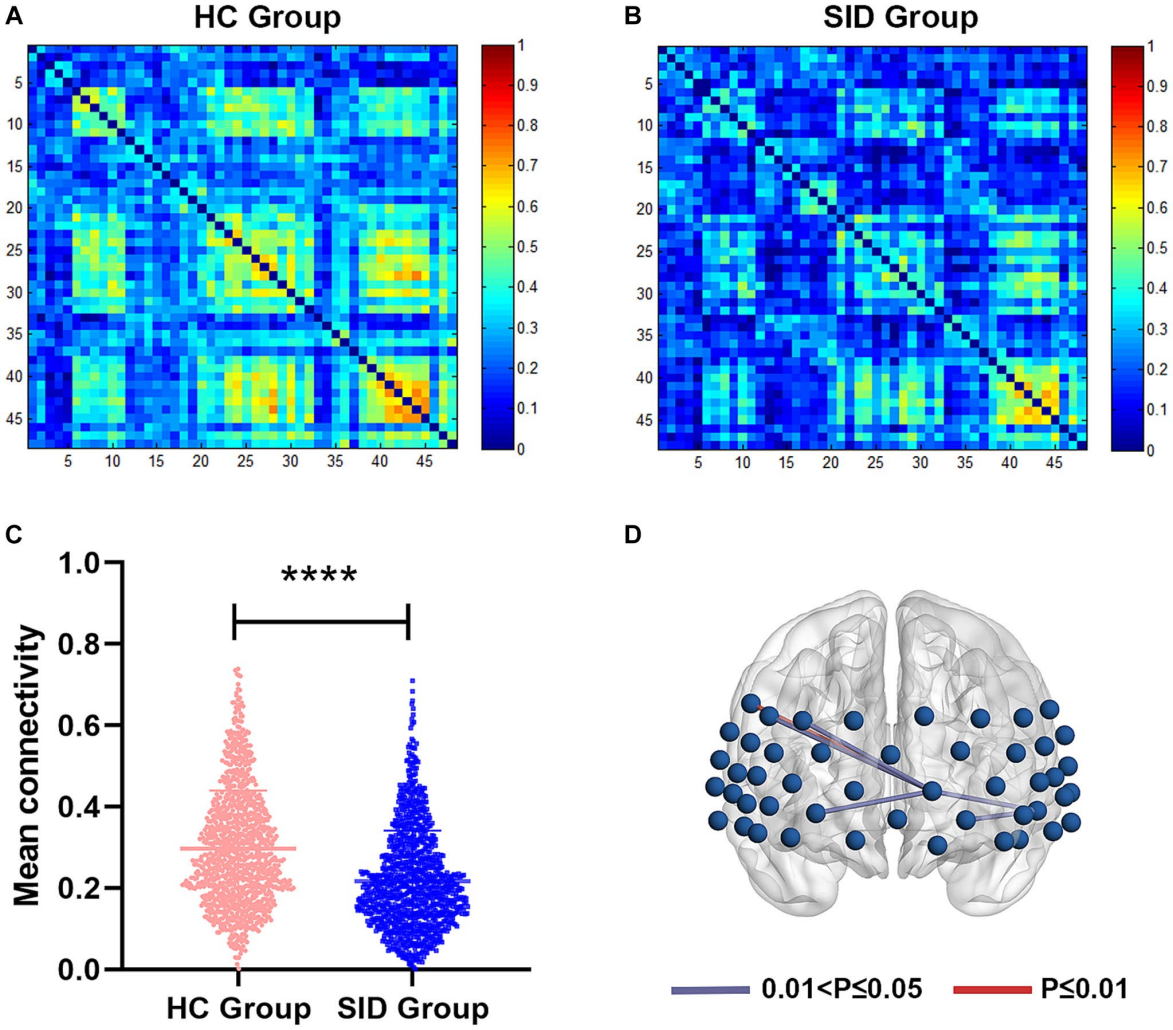
Functional connectivity between two groups. **(A,B)** The average functional connectivity strength of 48 channels in HC group and SID group, respectively. **(C)** Comparisons of mean connectivity strength of 48 channels between two groups. **(D)** Six channel pairs had significantly higher connectivity in HC group than SID group (FDR corrected *p* < 0.05). HC, healthy control; SID, short-term insomnia disorder. ****p < 0.0001.

Through FC analysis, two oxy-Hb correlation matrix maps were obtained for the SID group and HC group (Figures 2A,B). The mean strength of channel-to-channel connectivity was found to be significantly lower in the SID group compared to the HC group (0.217 ± 0.125 vs. 0.297 ± 0.142, *p* < 0.0001; Figure 2C). Furthermore, as shown in Figure 2D, the SID group exhibited significantly decreased FC strength in six pairs of channels compared to the HC group: Ch8-Ch27, Ch11-Ch14, Ch14-Ch27, Ch27-Ch38, Ch27-Ch39, and Ch27-Ch41 (*p* < 0.05, FDR corrected). Notably, channel 27 (corresponding to the left frontopolar cortex) appeared most frequently (5 times) in the channels displaying differential connectivity. Conversely, no channel pair with significantly higher connectivity was observed in the SID group. Please refer to Table 2 for detailed information regarding the differential channel pairs and their corresponding brain regions.

**Table 2:** Channel pairs with significant differences in connectivity strength between groups (mean ± SD).

Channel pairs	Brain regions	Connectivity strength		*t*	*p* (FDR corrected)
		SID group	HC group		
Ch8-Ch27	RFPC-LFPC	0.15 ± 0.39	0.52 ± 0.34	3.968	0.044
Ch11-Ch14	LOFC-LTPC	−0.02 ± 0.35	0.34 ± 0.35	4.136	0.044
Ch14-Ch27	LTPC-LFPC	0.01 ± 0.37	0.37 ± 0.34	3.908	0.044
Ch27-Ch38	LFPC-RPMC/SMA	0.03 ± 0.43	0.50 ± 0.32	4.923	0.007
Ch27-Ch39	LFPC-RDPFC	0.22 ± 0.34	0.52 ± 0.26	3.964	0.044
Ch27-Ch41	LFPC-RDPFC	0.18 ± 0.40	0.54 ± 0.31	3.946	0.044

SD, standard deviation; SID, short-term insomnia disorder; HC, healthy control; RFPC, right frontopolar cortex; LFPC, left frontopolar cortex; LOFC, left orbitofrontal cortex; LTPC, left temporopolar cortex; RPMC/SMA, right pre-motor and supplementary motor cortex; RDPFC, right dorsolateral prefrontal cortex; FDR, false discovery rate.

### 3.3. Correlations with clinical characteristics

**Figure 3:**
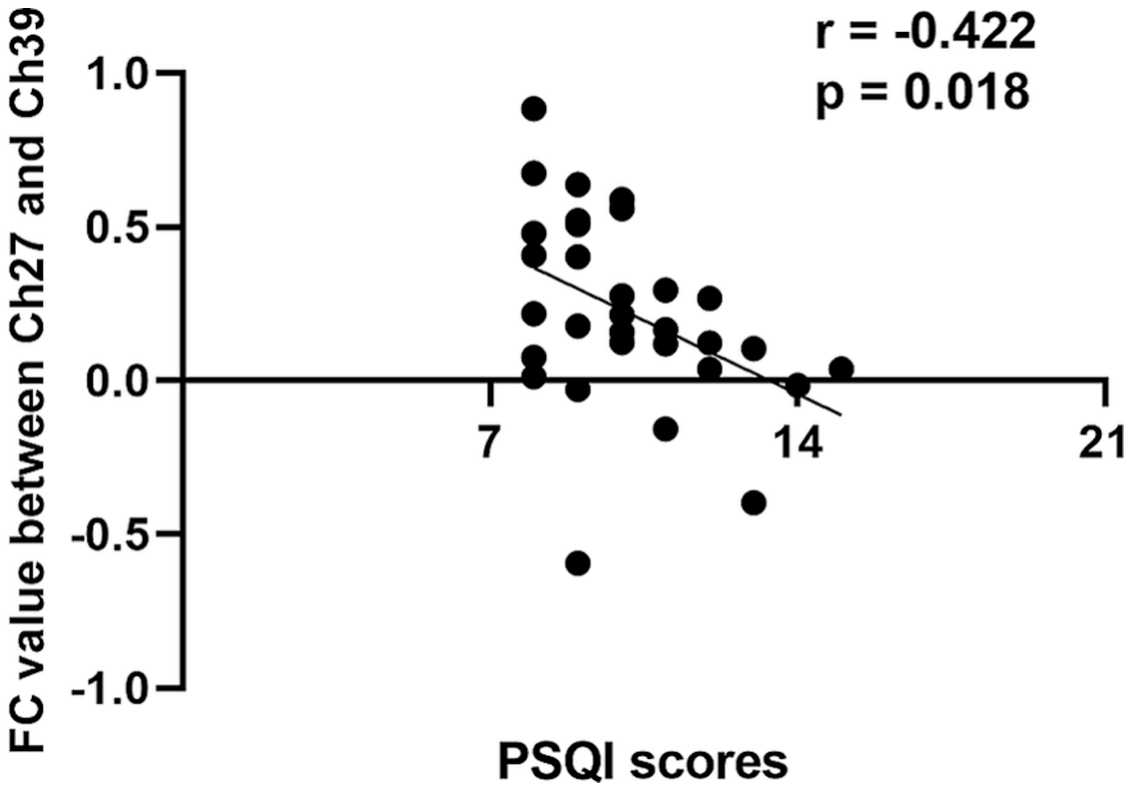
Correlation analysis between channel pair Ch27-Ch39 functional connectivity values and PSQI scores in short-term insomnia disorder group. FC, functional connectivity; Ch, channel; PSQI, Pitsburgh sleep quality index.

The FC values of the differential channel pairs for each participant were extracted and subjected to a Pearson correlation analysis with PSQI scores. As shown in Figure 3, the channel pair Ch27-Ch39, representing the left frontopolar cortex (LFPC) and right dorsolateral prefrontal cortex (RDPFC), exhibited a significant negative correlation with PSQI scores (r = −0.422, *p* = 0.018). This finding suggested that decreased connectivity between the LFPC and the RDPFC is associated with higher PSQI scores and poorer sleep quality.

## 4. Discussion

In order to unravel the pathophysiological mechanisms underlying short-term insomnia disorder, the present study utilized fNIRS to investigate the hemodynamic response and functional connectivity characteristics in the prefrontal cortex of patients with SID during VFT task. Furthermore, we observed whether these changes were correlated with poor sleep quality. To our knowledge, this study represents the first report to employ fNIRS combined with a cognitive task in the SID patients.

No significant differences in terms of integral values, centroid values, and mean Oxy-Hb variations were found between the SID group and the HC group in the VFT task, suggesting similar hemodynamic responses in both groups. However, our findings contrast with those of a previous study that reported significantly hypoactivated frontopolar cortex and right dorsolateral prefrontal cortex in individuals with chronic insomnia during the VFT task (Gong et al., 2022). This disparity may be attributed to variations in the severity of insomnia symptoms (as measured by PSQI scores: 16.1 ± 2.4 vs. 10.03 ± 1.97) and the duration of insomnia (25.4 ± 23.2 months vs. 1.88 ± 0.69 months) within the populations under investigation. This could be due to the fact that SID has a relatively smaller impact on brain activation patterns compared to chronic insomnia, which likely accounts for the similar performance of individuals with SID and healthy controls during the VFT task.

Insomnia has been recognized as a disorder associated with abnormal brain network connectivity (Nardone et al., 2020). Through functional connectivity analysis, we observed a notable decrease in connection strength between the left frontopolar cortex (LFPC) and several other brain regions in patients with SID. These regions include the right frontopolar cortex (RFPC), left temporopolar cortex (LTPC), right pre-motor and supplementary motor cortex (RPMC/SMA), and right dorsolateral prefrontal cortex (RDPFC). Similar to our research findings, Mukli et al. (2021) utilized fNIRS technology to investigate changes in prefrontal functional brain networks in 10 healthy young males following sleep deprivation, and reported a significant decrease in the number of functional connections in the overall network response. Additional investigations conducted using fNIRS (Bu et al., 2017), functional magnetic resonance imaging (fMRI; Wang et al., 2022) and electroencephalography (EEG; Verweij et al., 2014) have also reported diminished connectivity in the prefrontal cortex after sleep deprivation. These findings suggest that even short-term sleep disorder can exert adverse effects on the synchronized interactions among brain regions. The dorsolateral prefrontal cortex is a crucial region for maintaining working memory and executive function (Vartanian et al., 2014). The RPMC/SMA and temporopolar cortex also play a crucial role in executive function (Xu et al., 2020; Godefroy et al., 2023). The functional connectivity abnormalities in these regions may indicate cognitive decline. In turn, it is possible that one of the functions of sleep is to autonomously regulate the connectivity of the prefrontal brain network, thereby optimizing cognitive performance.

Evidence derived from studies conducted on non-human primates suggests that the FPC possesses various outgoing connections, such as those with the cingulate gyrus and superior temporal gyrus, as well as incoming connections from regions like the amygdala, thalamus, and basal forebrain (Petrides and Pandya, 2007; Burman et al., 2011). These connections provide support for the proposition that the FPC serves as a vital hub for integrating cognitive processes across multiple domains. In this study, we observed notable reduction in connectivity between the FPC and the other brain regions in SID patients. This finding suggest that lack of sleep may disrupt the functional network of the FPC, potentially leading to difficulties in maintaining normal cognitive activities.

Furthermore, our study revealed a negative correlation between FC values of the LFPC and the RDPFC with PSQI scores. This suggests that decreased connectivity between the LFPC and the RDPFC is associated with poorer sleep quality. Consistent with previous findings, patients with SID exhibit reduced activation in the FPC and the RDPFC during verbal fluency tasks, indicating a potential relationship between dysfunction in these regions and sleep disorders. Additionally, Hermesdorf et al. (2021) has observed that arousal might interfere with the transition to quiet slow-wave sleep and lead to a reduction in the volume of gray matter in the FPC. Therefore, it is plausible that the FPC not only plays a role in cognitive processes but also is involved in sleep maintenance.

The RDPFC has also been implicated in regulating normal sleep patterns. Jiang et al. (2013) used low-frequency repetitive transcranial magnetic stimulation (rTMS) to stimulate the dorsolateral prefrontal cortex (DLPFC) in the treatment of patients with SID and found significant improvements in stage III sleep and REM sleep cycles. One possible mechanism is that low-frequency rTMS stimulation of the DLPFC promotes the secretion of melatonin, brain serotonin, and norepinephrine, thereby maintaining normal sleep–wake cycles. We hypothesize that the decline in functional connectivity between the FPC and the RDPFC may lead to a reduction in reduced secretion of sleep-regulating hormones, ultimately resulting in the overactivation of the hypothalamic–pituitary–adrenal (HPA) and hypothalamic–pituitary-thyroid (HPT) axes, consequently causing a hyperarousal state in insomnia patients. Restoring this abnormal functional connection may help decrease arousal levels, improve sleep quality, and enhance daytime functioning. However, further investigation is required to determine the precise mechanisms through which these abnormal functional connectivity patterns contribute to sleep disorders.

This study utilizes fNIRS technology to evaluate the prefrontal cortex function in individuals diagnosed with SID, aiming to obtain a more comprehensive comprehension of the neural mechanisms and biomarkers implicated in insomnia. The utilization of fNIRS offers valuable insights into the underlying pathophysiological processes associated with insomnia, thereby facilitating the development of more precise and dependable diagnostic and assessment tools. The abnormal prefrontal pathways identified in this study could potentially serve as new targets for further transcranial stimulation treatments. Future research could validate the effectiveness of these new targets, providing information for the development of personalized treatment approaches.

### 4.1. Limitations

Several limitations in our study should be acknowledged. Firstly, participant selection was solely based on subjective questionnaires, which may have introduced bias, as objective parameters such as polysomnography data were not included. Secondly, the absence of significant differences in brain activation patterns between the two groups may be attributed to the small sample size. Thus, future studies should consider expanding the sample size to enhance the robustness of our findings. Thirdly, it is important to note that fNIRS technology only measures cortical activity on the surface and is unable to detect subcortical structures that are beyond the reach of near-infrared light. Hence, combining fNIRS with complementary imaging techniques such as EEG and MRI would improve both the temporal and spatial resolution of our investigations. Fourthly, considering that SID may impact multiple cognitive domains, it would be beneficial to include other assessments in addition to the VFT task in future studies to achieve a more comprehensive cognitive evaluation.

## 5. Conclusion

Patients with short-term insomnia disorder showed aberrant functional connectivity pattern in the prefrontal cortex during VFT task. Notably, the disruption in connectivity strength between the LFPC and the RDPFC was found to be significantly correlated with the severity of sleep disturbances. By employing fNIRS technology, it becomes possible to detect and diagnose individuals with SID at an early stage, thereby mitigating the risk of disease progression effectively.

## Data availability statement

The raw data supporting the conclusions of this article will be made available by the authors, without undue reservation.

## Ethics statement

The studies involving humans were approved by Ethics Committee of Jiangbin Hospital of Guangxi Zhuang Autonomous Region. The studies were conducted in accordance with the local legislation and institutional requirements. Written informed consent for participation in this study was provided by the participants’ legal guardians/next of kin.

## Author contributions

PW: Conceptualization, Formal analysis, Methodology, Visualization, Writing – original draft. CW: Data curation, Investigation, Writing – original draft. MW: Data curation, Investigation, Writing – original draft. YL: Methodology, Software, Writing – original draft. YX: Data curation, Investigation, Writing – original draft. XL: Resources, Writing – original draft. JJ: Writing – original draft, Resources. YB: Investigation, Software, Writing – original draft. JD: Writing – review & editing, Resources. WJ: Conceptualization, Funding acquisition, Writing – review & editing.
