# Supplementary material for: Prefrontal cortex functional connectivity changes during verbal fluency test in adults with short-term insomnia disorder: a functional near-infrared spectroscopy study
Source: Front Neurosci. 2023 Nov 9;17:1277690. doi: 10.3389/fnins.2023.1277690 (PMC10665481; doi:10.3389/fnins.2023.1277690)
Supplement: Supplementary file 1 [file Table_1.DOCX]

**Supplementary Table 1** The corresponding brain regions of 48 NIRS channels.

| Label of Channel | Brodmann Area | Percentage |
| --- | --- | --- |
| CH1 (S1-D1) | 21 - Middle Temporal gyrus | 0.9655 |
|  | 22 - Superior Temporal Gyrus | 0.0345 |
|  |  |  |
| CH2 (S1-D6) | 21 - Middle Temporal gyrus | 0.6087 |
|  | 22 - Superior Temporal Gyrus | 0.3913 |
|  |  |  |
| CH3 (S2-D1) | 21 - Middle Temporal gyrus | 0.2043 |
|  | 38 - Temporopolar area | 0.7778 |
|  | 48 - Retrosubicular area | 0.0179 |
|  |  |  |
| CH4 (S2-D2) | 38 - Temporopolar area | 0.0241 |
|  | 45 - pars triangularis Broca's area | 0.2234 |
|  | 46 - Dorsolateral prefrontal cortex | 0.3265 |
|  | 47 - Inferior prefrontal gyrus | 0.4261 |
|  |  |  |
| CH5 (S2-D7) | 38 - Temporopolar area | 0.3192 |
|  | 44 - pars opercularis_ part of Broca's area | 0.0033 |
|  | 45 - pars triangularis Broca's area | 0.5407 |
|  | 48 - Retrosubicular area | 0.1368 |
|  |  |  |
| CH6 (S3-D2) | 10 - Frontopolar area | 0.2828 |
|  | 11 - Orbitofrontal area | 0.1148 |
|  | 46 - Dorsolateral prefrontal cortex | 0.2787 |
|  | 47 - Inferior prefrontal gyrus | 0.3238 |
|  |  |  |
| CH7 (S3-D3) | 10 - Frontopolar area | 0.1629 |
|  | 11 - Orbitofrontal area | 0.8371 |
|  |  |  |
| CH8 (S3-D8) | 10 - Frontopolar area | 0.5548 |
|  | 11 - Orbitofrontal area | 0.4452 |
|  |  |  |
| CH9 (S4-D3) | 10 - Frontopolar area | 0.011 |
|  | 11 - Orbitofrontal area | 0.989 |
|  |  |  |
| CH10 (S4-D4) | 10 - Frontopolar area | 0.099 |
|  | 46 - Dorsolateral prefrontal cortex | 0.5198 |
|  | 47 - Inferior prefrontal gyrus | 0.3812 |
|  |  |  |
| CH11 (S4-D9) | 10 - Frontopolar area | 0.4324 |
|  | 11 - Orbitofrontal area | 0.5676 |
|  |  |  |
| CH12 (S5-D4) | 38 - Temporopolar area | 0.9897 |
|  | 47 - Inferior prefrontal gyrus | 0.0103 |
|  |  |  |
| CH13 (S5-D5) | 21 - Middle Temporal gyrus | 0.9762 |
|  | 38 - Temporopolar area | 0.0238 |
|  |  |  |
| CH14 (S5-D10) | 21 - Middle Temporal gyrus | 0.0341 |
|  | 38 - Temporopolar area | 0.6177 |
|  | 48 - Retrosubicular area | 0.3481 |
|  |  |  |
| CH15 (S6-D5) | 20 - Inferior Temporal gyrus | 0.1467 |
|  | 21 - Middle Temporal gyrus | 0.8533 |
|  |  |  |
| CH16 (S6-D11) | 20 - Inferior Temporal gyrus | 0.0955 |
|  | 21 - Middle Temporal gyrus | 0.6688 |
|  | 22 - Superior Temporal Gyrus | 0.2357 |
|  |  |  |
| CH17 (S7-D1) | 6 - Pre-Motor and Supplementary Motor Cortex | 0.0271 |
|  | 21 - Middle Temporal gyrus | 0.2508 |
|  | 22 - Superior Temporal Gyrus | 0.0678 |
|  | 38 - Temporopolar area | 0.0678 |
|  | 48 - Retrosubicular area | 0.5864 |
|  |  |  |
| CH18 (S7-D6) | 22 - Superior Temporal Gyrus | 0.5314 |
|  | 43 - Subcentral area | 0.3805 |
|  | 48 - Retrosubicular area | 0.0881 |
|  |  |  |
| CH19 (S7-D7) | 6 - Pre-Motor and Supplementary Motor Cortex | 0.4277 |
|  | 44 - pars opercularis_ part of Broca's area | 0.2862 |
|  | 45 - pars triangularis Broca's area | 0.0193 |
|  | 48 - Retrosubicular area | 0.2669 |
|  |  |  |
| CH20 (S7-D12) | 4 - Primary Motor Cortex | 0.0671 |
|  | 6 - Pre-Motor and Supplementary Motor Cortex | 0.3706 |
|  | 43 - Subcentral area | 0.5623 |
|  |  |  |
| CH21 (S8-D2) | 45 - pars triangularis Broca's area | 0.1522 |
|  | 46 - Dorsolateral prefrontal cortex | 0.8478 |
|  |  |  |
| CH22 (S8-D7) | 45 - pars triangularis Broca's area | 0.9792 |
|  | 46 - Dorsolateral prefrontal cortex | 0.0208 |
|  |  |  |
| CH23 (S8-D8) | 10 - Frontopolar area | 0.4431 |
|  | 46 - Dorsolateral prefrontal cortex | 0.5569 |
|  |  |  |
| CH24 (S8-D13) | 45 - pars triangularis Broca's area | 0.8 |
|  | 46 - Dorsolateral prefrontal cortex | 0.2 |
|  |  |  |
| CH25 (S9-D3) | 10 - Frontopolar area | 0.8875 |
|  | 11 - Orbitofrontal area | 0.1125 |
|  |  |  |
| CH26 (S9-D8) | 10 - Frontopolar area | 1 |
|  |  |  |
| CH27 (S9-D9) | 10 - Frontopolar area | 1 |
|  |  |  |
| CH28 (S9-D14) | 10 - Frontopolar area | 1 |
|  |  |  |
| CH29 (S10-D4) | 45 - pars triangularis Broca's area | 0.3285 |
|  | 46 - Dorsolateral prefrontal cortex | 0.6715 |
|  |  |  |
| CH30 (S10-D9) | 10 - Frontopolar area | 0.3909 |
|  | 46 - Dorsolateral prefrontal cortex | 0.6091 |
|  |  |  |
| CH31 (S10-D10) | 45 - pars triangularis Broca's area | 0.9964 |
|  | 48 - Retrosubicular area | 0.0036 |
|  |  |  |
| CH32 (S10-D15) | 45 - pars triangularis Broca's area | 0.9016 |
|  | 46 - Dorsolateral prefrontal cortex | 0.0984 |
|  |  |  |
| CH33 (S11-D5) | 21 - Middle Temporal gyrus | 0.5385 |
|  | 22 - Superior Temporal Gyrus | 0.4114 |
|  | 48 - Retrosubicular area | 0.0502 |
|  |  |  |
| CH34 (S11-D10) | 6 - Pre-Motor and Supplementary Motor Cortex | 0.2691 |
|  | 22 - Superior Temporal Gyrus | 0.0532 |
|  | 43 - Subcentral area | 0.093 |
|  | 48 - Retrosubicular area | 0.5847 |
|  |  |  |
| CH35 (S11-D11) | 21 - Middle Temporal gyrus | 0.2033 |
|  | 22 - Superior Temporal Gyrus | 0.7967 |
|  |  |  |
| CH36 (S11-D16) | 2 - Primary Somatosensory Cortex | 0.0627 |
|  | 22 - Superior Temporal Gyrus | 0.0941 |
|  | 43 - Subcentral area | 0.7247 |
|  | 48 - Retrosubicular area | 0.1185 |
|  |  |  |
| CH37 (S12-D7) | 44 - pars opercularis_ part of Broca's area | 0.4164 |
|  | 45 - pars triangularis Broca's area | 0.5836 |
|  |  |  |
| CH38 (S12-D12) | 4 - Primary Motor Cortex | 0.0078 |
|  | 6 - Pre-Motor and Supplementary Motor Cortex | 0.6163 |
|  | 9 - Dorsolateral prefrontal cortex | 0.0775 |
|  | 44 - pars opercularis_ part of Broca's area | 0.2984 |
|  |  |  |
| CH39 (S12-D13) | 9 - Dorsolateral prefrontal cortex | 0.0945 |
|  | 44 - pars opercularis_ part of Broca's area | 0.3425 |
|  | 45 - pars triangularis Broca's area | 0.4882 |
|  | 46 - Dorsolateral prefrontal cortex | 0.0748 |
|  |  |  |
| CH40 (S13-D8) | 9 - Dorsolateral prefrontal cortex | 0.0164 |
|  | 10 - Frontopolar area | 0.4098 |
|  | 46 - Dorsolateral prefrontal cortex | 0.5738 |
|  |  |  |
| CH41 (S13-D13) | 9 - Dorsolateral prefrontal cortex | 0.4519 |
|  | 45 - pars triangularis Broca's area | 0.0144 |
|  | 46 - Dorsolateral prefrontal cortex | 0.5337 |
|  |  |  |
| CH42 (S13-D14) | 9 - Dorsolateral prefrontal cortex | 0.8594 |
|  | 10 - Frontopolar area | 0.1205 |
|  | 46 - Dorsolateral prefrontal cortex | 0.0201 |
|  |  |  |
| CH43 (S14-D9) | 9 - Dorsolateral prefrontal cortex | 0.0041 |
|  | 10 - Frontopolar area | 0.4426 |
|  | 46 - Dorsolateral prefrontal cortex | 0.5533 |
|  |  |  |
| CH44 (S14-D14) | 9 - Dorsolateral prefrontal cortex | 0.8975 |
|  | 10 - Frontopolar area | 0.1025 |
|  |  |  |
| CH45 (S14-D15) | 9 - Dorsolateral prefrontal cortex | 0.4279 |
|  | 45 - pars triangularis Broca's area | 0.0047 |
|  | 46 - Dorsolateral prefrontal cortex | 0.5674 |
|  |  |  |
| CH46 (S15-D10) | 6 - Pre-Motor and Supplementary Motor Cortex | 0.2587 |
|  | 44 - pars opercularis_ part of Broca's area | 0.6049 |
|  | 45 - pars triangularis Broca's area | 0.1364 |
|  |  |  |
| CH47 (S15-D15) | 44 - pars opercularis_ part of Broca's area | 0.5194 |
|  | 45 - pars triangularis Broca's area | 0.4806 |
|  |  |  |
| CH48 (S15-D16) | 3 - Primary Somatosensory Cortex | 0.0189 |
|  | 4 - Primary Motor Cortex | 0.178 |
|  | 6 - Pre-Motor and Supplementary Motor Cortex | 0.5455 |
|  | 43 - Subcentral area | 0.2576 |
